# Edge-Enhanced Object-Space Model Optimization of Tomographic Reconstructions for Additive Manufacturing

**DOI:** 10.3390/mi14071362

**Published:** 2023-06-30

**Authors:** Yanchao Zhang, Minzhe Liu, Hua Liu, Ce Gao, Zhongqing Jia, Ruizhan Zhai

**Affiliations:** 1 Changchun Institute of Optics, Fine Mechanics and Physics, Chinese Academy of Sciences (CAS), Changchun 130033, China; zhangyanchaomn@126.com; 2 Laser Institute, Qilu University of Technology (Shandong Academy of Sciences), Qingdao 266000, China; minzheliu@sdlaser.cn (M.L.); jiazhongqing@vip.sdlaser.cn (Z.J.); zrz@vip.sdlaser.cn (R.Z.); 3 Center for Advanced Optoelectronic Functional Materials Research, and Key Laboratory for UV Emitting Materials and Technology of Ministry of Education, National Demonstration Center for Experimental Physics Education, Northeast Normal University, Changchun 130024, China; liuh146@nenu.edu.cn

**Keywords:** volumetric additive manufacturing, tomographic reconstruction, optimization, OSMO, edge enhanced

## Abstract

Object-space model optimization (OSMO) has been proven to be a simple and high-accuracy approach for additive manufacturing of tomographic reconstructions compared with other approaches. In this paper, an improved OSMO algorithm is proposed in the context of OSMO. In addition to the two model optimization steps in each iteration of OSMO, another two steps are introduced: one step enhances the target regions’ in-part edges of the intermediate model, and the other step weakens the target regions’ out-of-part edges of the intermediate model to further improve the reconstruction accuracy of the target boundary. Accordingly, a new quality metric for volumetric printing, named ‘Edge Error’, is defined. Finally, reconstructions on diverse exemplary geometries show that all the quality metrics, such as VER, PW, IPDR, and Edge Error, of the new algorithm are significantly improved; thus, this improved OSMO approach achieves better performance in convergence and accuracy compared with OSMO.

## 1. Introduction

**Figure 1:**
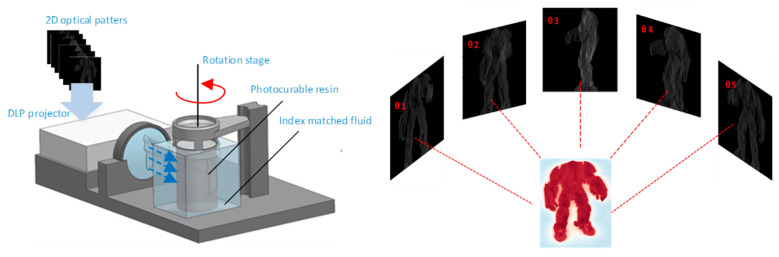
Overview of tomographic VAM.

Volumetric additive manufacturing (VAM) has introduced a significant improvement in the development of 3D printing in recent years because of its low surface roughness and high printing efficiency. Tomographic VAM, as the major VAM implementation, was derived from computed tomography (CT) and the Fourier slice theorem. In tomographic VAM printing, a series of 2D optical patterns (known as image sets) are projected onto a rotating volume, which is filled with photosensitive liquid resin. Then, the expected 3D structure is polymerized in seconds to minutes and removed from the remaining liquid resin, as shown in Figure 1.

The progress of VAM technology depends on three elements: optics, material science, and image computation. Many studies have been reported on the first two elements [1,2,3,4,5,6,7,8,9,10,11], while research on image computation, especially iterative image optimization of the inverse math problem, has just begun [12,13,14].

**Figure 2:**
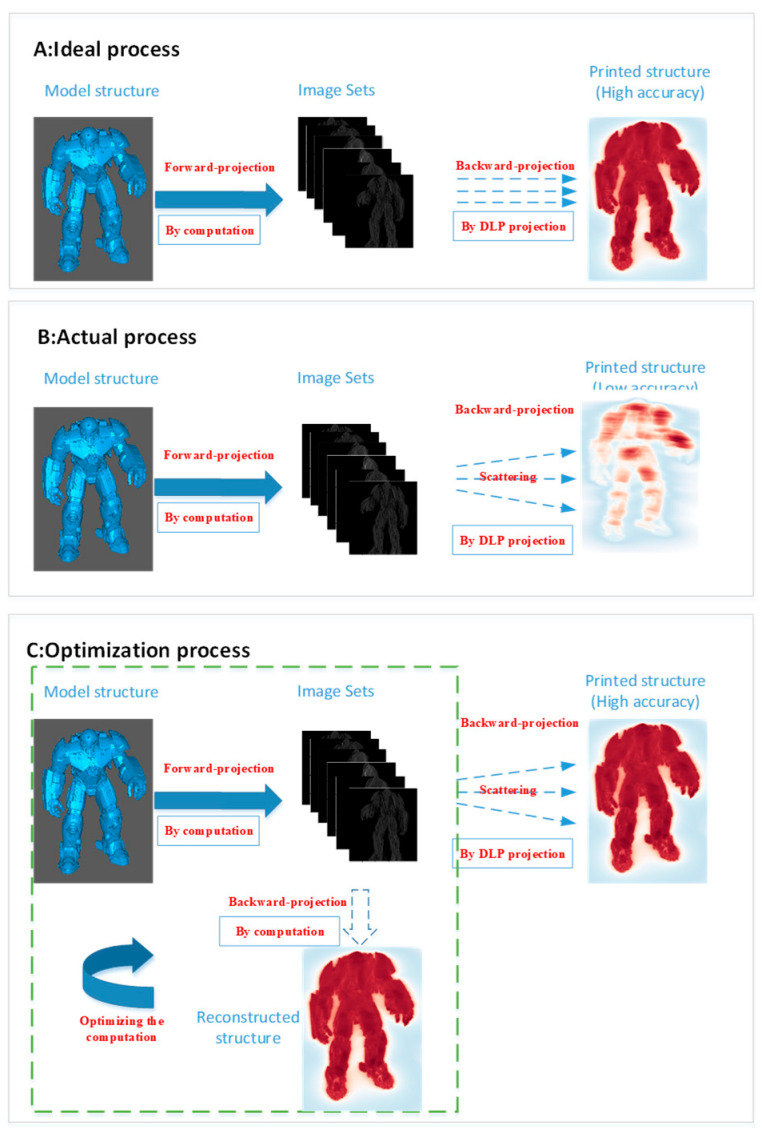
Image computation process.

Derived from computed tomography (CT) and the Fourier slice theorem, the ideal process of tomographic VAM is to obtain image sets from the model structure by forward-projection computing and then to obtain the printed structure by digital light processing (DLP) projection [2,3] (backward projection), as shown in Figure 2A. However, we can only receive a low-accuracy printed structure due to light scattering, as shown in Figure 2B. Consequently, it is necessary to optimize the computation before DLP projection to obtain a structure with higher accuracy, as shown in Figure 2C.

Light scattering causes it to be unable to propagate along a straight line. Therefore, some computation methods, such as FBP, gradient-descent optimization, and OSMO, by frequency filtering or optimization iteration, are used to approximate the printed structure to the model structure, so as to improve printing accuracy. The relevant optimization algorithms mentioned in this paper are all discussed based on this premise.

The well-known filtered back projection (FBP) method, constrained by its negative results, can only produce projector images with low accuracy, while the intensity of these images must be non-negative [15,16].

Other approaches that use gradient-descent optimization to adjust image sets to improve the volumetric dose reconstruction indirectly have been proposed [12,13,14]. Different from the above approaches, OSMO is applied to optimize the structure directly, instead of optimizing the set of external projection images, and can achieve better printing accuracy [13]. In addition, OSMO has two other excellent advantages: flexibility and ease of implementation and use.

In this paper, an improved OSMO algorithm is proposed in the context of OSMO, named ‘edge-enhanced OSMO’. The low surface roughness of a print target depends on the accurate reconstruction of the target boundary. Therefore, some morphological processing is imported into OSMO to enhance the target regions’ in-part edges of the intermediate model and weaken its out-of-part edges to achieve better performance in convergence and accuracy compared with OSMO. Accordingly, a new quality metric for volumetric printing is defined to evaluate the accuracy of the reconstructed target boundary as a supplement to other quality metrics.

## 2. OSMO Algorithm for VAM

To describe the OSMO algorithm, we use mathematical notation consistent with that of Rackson and Champley et al. [13]. The desired geometry to print is denoted as f_T_. The forward-projection operator that transforms an object to an image set is denoted as P. The notation is consistent with the fact that it performs the mathematical inverse transformation of the forward projection, which is denoted as P*. Therefore, Pf_T_ is the forward projection of f_T_ from the object to the image set. Let N be a normalizing operator. *D_l_* and *D_h_* are defined as the low-dose threshold value and high-dose threshold value, respectively, where 0 < *D_l_* < *D_h_* <1. Here, *M_j,j_* is the object model after j iterations. M_0,0_ is defined as the initial model of *M_j,j_*, and its value is set to f_T_. We define f_j,j_ as the forward projection of *M_j,j_*, any resultant negative values of which are set to zero. The expression of f_j,j_ is as follows:

(1) $$f_{j,j}={\;\mathrm{NP}}^{*}\max\left(0,{\mathrm{PM}}_{j,j}\right),$$

**Figure 3:**
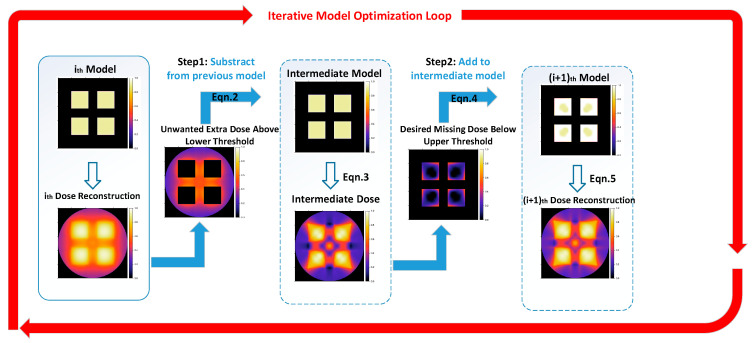
OSMO optimization process (mention modified from [13]).

The OSMO iteration process for optimizing from *M_j,j_* to *M_j_*_+1,*j*+1_ is shown in Figure 3, and it includes the following two steps:

**STEP 1**: Update the intermediate model *M_i,i_*_+1_ by subtracting out-of-part voxels with an unwanted extra dose above the lower threshold *D_l_* from the previous model *M_i,i._* For only the out-of-part voxels (in-part voxels remain unchanged from *M_i,i_*):

(2) $$M_{i,i+1}=M_{i,i}-max\left(0,f_{i,i}-D_{l}\right).$$

According to Equation (1), the expression of the intermediate reconstruction $f_{i,i+1}$ is:

(3) $$f_{i,i+1}={\;\mathrm{NP}}^{*}\max\left(0,{\mathrm{PM}}_{i,i+1}\right).$$

**STEP 2**: Update the model *M_i_*_+1,*i*+1_ by adding in-part voxels with the desired missing dose below the upper threshold *D_h_* to the intermediate model *M_i,i_*_+1_. For only in-part voxels (out-of-part voxels remain unchanged from *M_i,i_*_+1_):

(4) $$M_{i+1,i+1}=M_{i,i+1}+max\left(0,D_{h}-f_{i,i+1}\right).$$

According to Equation (1), the expression of the reconstruction $f_{i+1,i+1}$ is

(5) $$f_{i+1,i+1}={\;\mathrm{NP}}^{*}\max\left(0,{\mathrm{PM}}_{i+1,i+1}\right).$$

Then, the image sets can be solved after K iterations, as shown in Equation (6):

(6) $$\mathrm{ImgS}\;=\;\mathrm{Nmax}\left(0,{\mathrm{PM}}_{K,K}\right).$$

## 3. Edge-Enhanced OSMO Algorithm for VAM

### 3.1. Edge-Enhanced OSMO Principle

**Figure 4:**
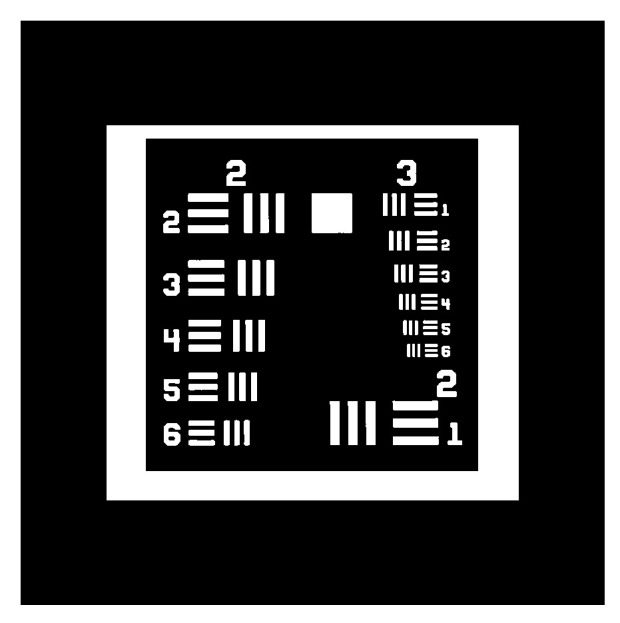
Binary target geometry of ‘Reschart’.

The direct criterion for evaluating the optimization quality of reconstruction algorithms is the degree of separation between the in-part and out-of-part edges in the histogram of the reconstructed image. The better the separation is, the less overlap there is, and the higher the reconstruction accuracy that will be obtained, and vice versa. For ease of explanation, 2D ‘Reschart’ (as shown in Figure 4) is taken as the example, which can be considered as one of the 3D geometry’s cross-sections.

**Figure 5:**
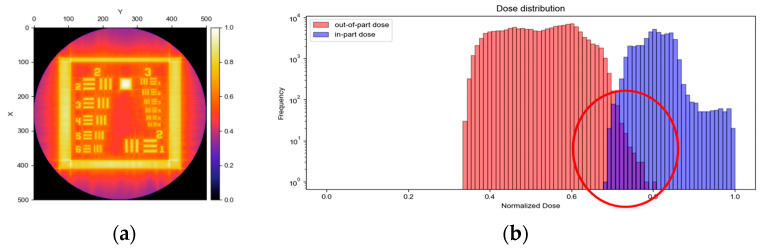
Dose distribution reconstruction results of ‘Reschart’ with OSMO (Iteration = 15, *D_l_* = 0.6, *D_h_* = 0.8): (**a**) dose distribution; (**b**) histogram of the dose distribution.

There is still a significant overlap between the histogram’s in-part and out-of-part edges after 15 optimization iterations (*D_h_* = 0.8, *D_l_* = 0.6) with OSMO (see Figure 5). Using these image sets corresponding to the above iteration result for printing, the expected printing accuracy will not be achieved. Therefore, in the case that the number of iterations is given, or the iteration accuracy is in a convergence state, we need to find ways to reduce the overlap area between the in-part and out-of-part edges to further improve printing accuracy.

**Figure 6:**
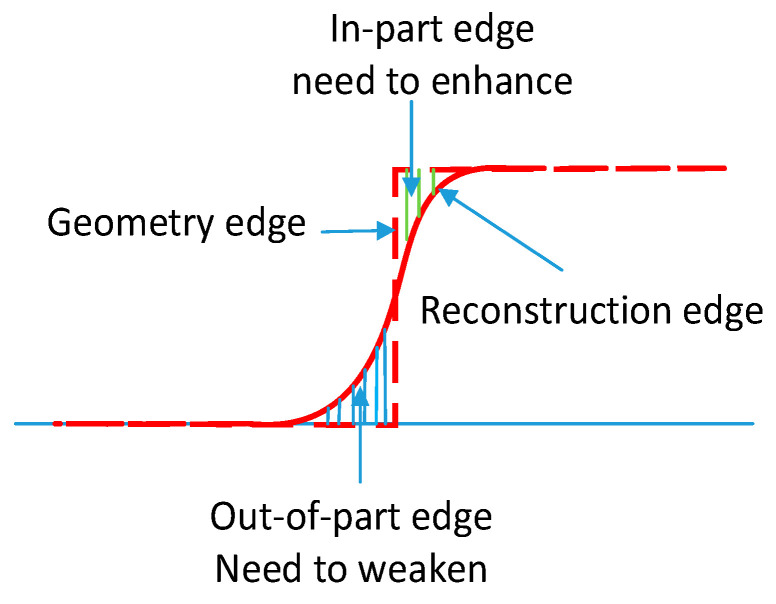
Edge enhancement schematic diagram.

In target geometry, the target’s boundaries (or edges) belong to the transition area between the in-part and out-of-part edges, which are the main part of the dose distribution histogram’s overlapping area, and the accurate reconstruction of edge regions is the key to improving printing accuracy. Therefore, it is necessary to enhance the in-part edge regions and weaken the out-of-part edge regions during the optimization iteration process, as shown in Figure 6, to further improve the contrast of the edge regions and promote better separation of the target and background.

To achieve the above purpose, there are generally two approaches: one is increasing the number of optimization iterations, and the other is enhancing the edge gradient.

The method of increasing the number of optimization iterations can improve the separation between the target and background to a certain extent. However, it belongs to global optimization. On the one hand, it is not possible to just focus on the optimization of edge regions, which may lead to the iteration falling into local convergence. On the other hand, it will increase the enormous but unnecessary computation of non-edge regions.

The method of enhancing edge gradients is easily thought of as frequency filtering. This method also has the same problem of global processing, which may increase unnecessary computational cost. In addition, the principle of frequency filtering is to increase or maintains the edges’ intensity, while weakening the intensity of the regions on both sides of the edges (including in-part edges and out-of-part edges) without distinction, and there is a certain deviation from the idea to ‘enhance the in-part edge regions and weaken the out-of-part edge regions’.

In this paper, the edge-enhanced OSMO algorithm (EE OSMO) is proposed, and it is an improved algorithm based on OSMO. First, a morphological method is used to extract in-part edges and out-of-part edges from the original target geometry. Then, the in-part edges are enhanced, and the out-of-part edges are weakened.

EE OSMO has the following two advantages: Firstly, based on the optimization concept of OSMO, local enhancement calculations are only performed on the edge regions, thus can achieve fast and high-precision convergence of the iteration with minimal additional operations. Secondly, compared with the problem of frequency filtering not being able to distinguish the two sides of the edge, the EE OSMO method enhances and weakens the edge regions according to their different regions, which can further improve the reconstruction accuracy of the edge regions.

Before introducing the algorithm implementation principle of EE OSMO, the extraction methods of in-part edges and out-of-part edges are explained.

**Figure 7:**
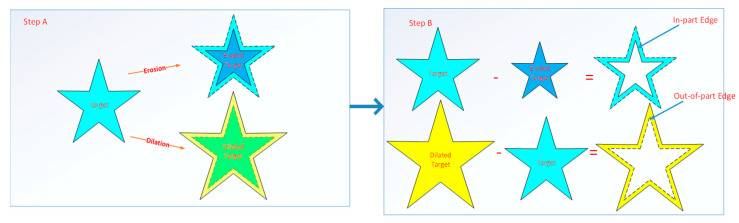
Schematic diagram of edge extraction.

The extraction process of in-part edges is as follows:

1. Erode the target regions of the geometry pattern, and then obtain the eroded target regions, as shown in the dark blue internal area in Figure 7 (Step A). The structural element used for erosion is the central symmetric structure (the size is 3 × 3 in this paper).
2. Subtract the eroded target regions from the target regions, and then obtain the in-part edges, as shown Figure 7 (Step B).

The extraction process of out-of-part edges is as follows:

1. Dilate the target regions of the geometry pattern, and then obtain the dilated target regions, as shown in the yellow internal area in Figure 7. Step A. The structural element used for dilation is the central symmetric structure (the size is 3 × 3 in this paper).
2. Subtract the target regions from the dilated target regions, and then obtain the out-of-part edges, as shown Figure 7 (Step B).

It should be noted that the extraction of in-part or out-of-part edges is an isotropic process; therefore, it is required that the structural elements used for erosion and dilation must be a central symmetric structure, and the unilateral size must be odd to ensure the same width of edge region in all directions. EE OSMO is a further correction to the reconstruction accuracy of OSMO, which only needs to enhance the edge area in a small range. The higher the size value, the greater the stiffness of the edge regions but the lower the accuracy. On the contrary, the smaller the stiffness of the edge region, the higher the accuracy. It is generally recommended to have a structural element size in the range of 3–7.

### 3.2. Edge-Enhanced OSMO Approach

**Figure 8:**
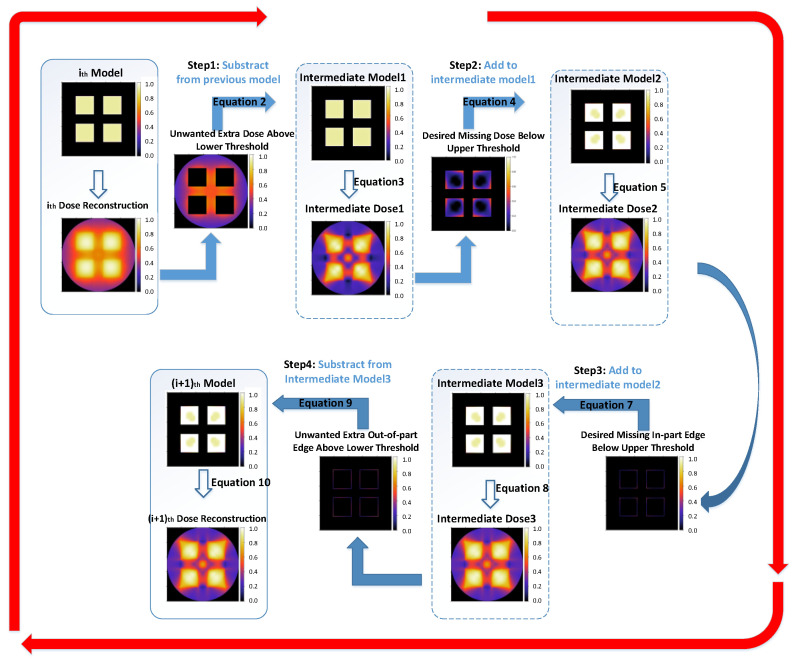
EE OSMO optimization process.

According to the principle described in the previous section, the implementation process of edge-enhanced OSMO (EE OSMO) is based on the OSMO two-step (STEP 1 and STEP 2) iterative method, and an additional two-step (STEP 3 and STEP 4) edge enhancement process is added, as shown in Figure 8. The implementation process is as follows:

**STEP 1**: Refer to Section 2 STEP 1 for details.

**STEP 2**: Refer to Section 2 STEP 2 for details.

**STEP 3**: Update intermediate Model 3 *M_i,i_*_+3_ by adding in-part edge voxels with the desired missing dose below the upper threshold *D_h_* to intermediate Model 2 *M_i,i_*_+2_. For only in-part edge voxels:

(7) $$M_{i,i+3}=M_{i,i+2}+max\left(0,D_{h}-f_{i,i+2}\right).$$

According to Equation (1), the expression of the reconstruction $f_{i,i+3}\;$is

(8) $$f_{i,i+3}={\;\mathrm{NP}}^{*}\max\left(0,{\mathrm{PM}}_{i,i+3}\right).$$

**STEP 4:** Update the model *M_i_*_+1,*i*+1_ by subtracting out-of-part edge voxels with an unwanted extra dose above the lower threshold *D_l_* from intermediate Model 3 *M_i,i_*_+3_. For only the out-of-part edge voxels:

(9) $$M_{i+1,i+1}=M_{i,i+3}-max\left(0,f_{i,i+3}-D_{l}\right).$$

According to Equation (1), the expression of the reconstruction $f_{i+1,i+1}$ is

(10) $$f_{i+1,i+1}={\;\mathrm{NP}}^{*}\max\left(0,{\mathrm{PM}}_{i+1,i+1}\right).$$

After N optimization iterations, the image sets can be achieved, as shown in Equation (6).

**Figure 9:**
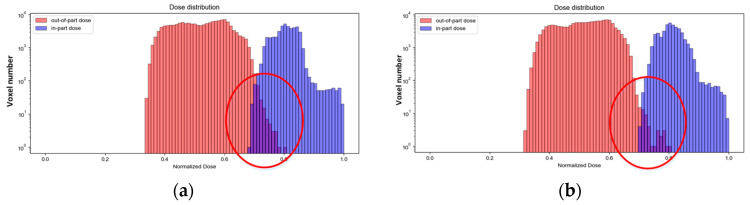
Dose distribution reconstruction histogram of ‘Reschart’ (Iteration = 15, *D_l_* = 0.6, *D_h_* = 0.8): (**a**) OSMO; (**b**) EE OSMO.

For the sake of comparison, the ‘Reschart’ image (as shown in Figure 3) is used as an example, and the reconstruction results of OSMO and EE OSMO are shown in Figure 9a,b, respectively, with 15 optimization iterations (*D_h_* = 0.8, *D_l_* = 0.6). The comparison graph in Figure 9 shows that the reconstruction accuracy of EE OSMO is significantly improved with the same parameters.

## 4. Reconstruction Quality Metrics

According to the literature [13], three metrics for evaluating the quality of a reconstruction are defined: Voxel Error Rate (VER), Process Window (PW) size, and In-part Dose Range (IPDR). As a supplement, a new quality metric named Edge Error (EdgeE) is proposed in order to evaluate the accuracy of the reconstruction’s edge regions.

The calculation of EdgeE consists of two parts: one is the in-part edge voxels error rate, that is, the in-part edge error rate (IPEER); the other is out-of-part edge voxels error rate, that is, the out-of-part edge error rate (OPEER). The expression for IPEER is

(11) $$\mathrm{IPEER}=\frac{\sum_{f_{i,i}\in R_{\mathrm{IPE}}}\mathrm{Sgn}(f_{i,i}<\mathrm{TH})}{N_{\mathrm{IPE}}},$$

where, $f_{i,i}$ is defined as the reconstruction dose distribution for the i-th iteration. $R_{\mathrm{IPE}}$ is defined as in-part edge regions. $N_{\mathrm{IPE}}$ is defined as the total number of in-part edge voxels. TH is the error threshold in the current iteration, and the expression is

(12) $$\mathrm{TH}=\left(\min\left(f_{i,i}\in R_{\mathrm{IP}}\right)+\max\left(f_{i,i}\in R_{\mathrm{OP}}\right)\right)/2,$$

where $R_{\mathrm{IP}}$ is defined as the in-part regions and $R_{\mathrm{OP}}$ as out-of-part regions. The expression for OPEER is

(13) $$\mathrm{OPEER}=\frac{\sum_{f_{i,i}\in R_{\mathrm{OPE}}}\mathrm{Sgn}(f_{i,i}>\mathrm{TH})}{N_{\mathrm{OPE}}},$$

where $R_{\mathrm{OPE}}$ is defined as out-of-part edge regions, and $N_{\mathrm{OPE}}$ is defined as total number of out-of-part edge voxels. The expression for EdgeE is

(14) EdgeE=(IPEER+OPEER)/2. 

The accuracy of the reconstruction’s edge regions determines the printing accuracy and surface smoothness. EdgeE, using the reconstruction accuracy of the target edges as the evaluation criterion, can intuitively characterize the reconstruction quality. It is a beneficial supplement to VER, PW, and IPDR.

## 5. Results and Discussion

### 5.1. Evaluation of Optimization

**Figure 10:**
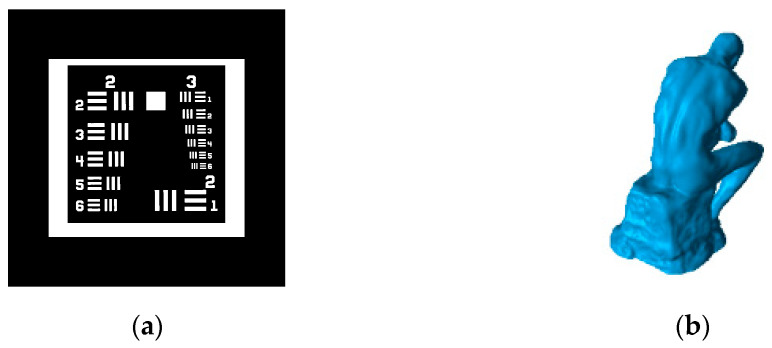
Geometric structures for evaluation: (**a**) target geometry of ‘Reschart’; (**b**) target geometry of ‘Thinker’.

Because EE OSMO is the improved algorithm for OSMO, two typical geometric structures derived from publicly available geometry files are selected as optimization reconstruction targets in this section to sufficiently evaluate the performance of EE OSMO. These geometries are descriptively titled ‘Reschart’ and ‘Thinker’, and the target geometries are shown in Figure 10. The performance of EE OSMO and OSMO are comprehensively compared using four quality metrics: VER, PW, IPDR, and EdgeE.

VER is defined as the voxel error rate. The closer the value of VER is to 0, the lower the error rate of printing.

PW is defined as the difference, in normalized units of dose, between the highest-dose out-of-part voxel and the lowest-dose in-part voxel [13]. There are two ways to define PW:

If PW is defined as

PW=(highest−dose out−of−part voxel)−(lowest−dose in−part voxel), it is negative when there are no overlaps between the in-part histogram and out-of-part histogram, and vice versa.

If PW is defined as

PW=(lowest−dose in−part voxel)−(highest−dose out−of−part voxel), it is positive when there are no overlaps between the in-part histogram and out-of-part histogram, and vice versa.

In this paper, the first definition of PW is used for algorithm evaluation.

IPDR is defined as the in-part dose range, which is one minus the lowest-dose in-part voxel. The closer the value of IPDR is to 0, the more concentrated the energy of the dose distribution.

EdgeE, as described in the previous section, is defined as the edges error rate. The closer the value of EdgeE is to 0, the lower the error rate of printing.

The optimization algorithm and all ancillary functions are implemented in Python3 using the python library VAMToolbox [17], which supports the generation of light projections and the control of a DLP projector for tomographic VAM. All functions are implemented in Visual Studio Code, which is available on request.

**Figure 11:**
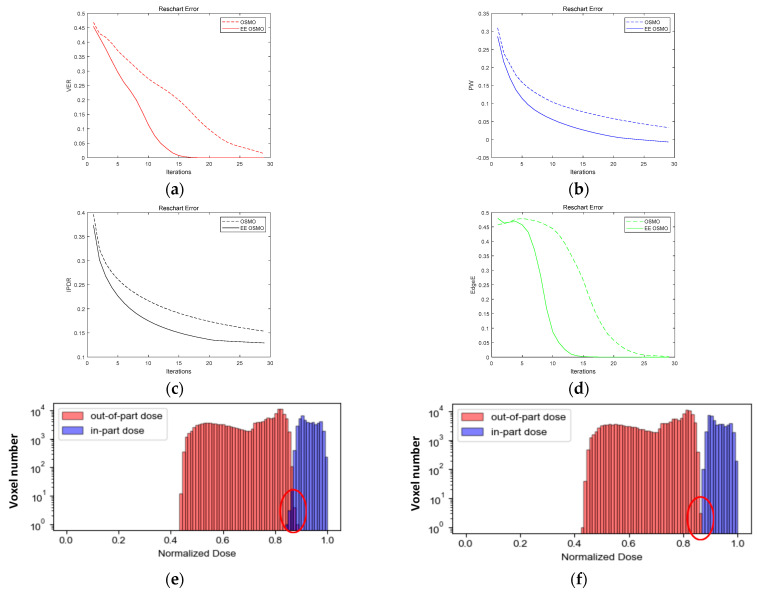

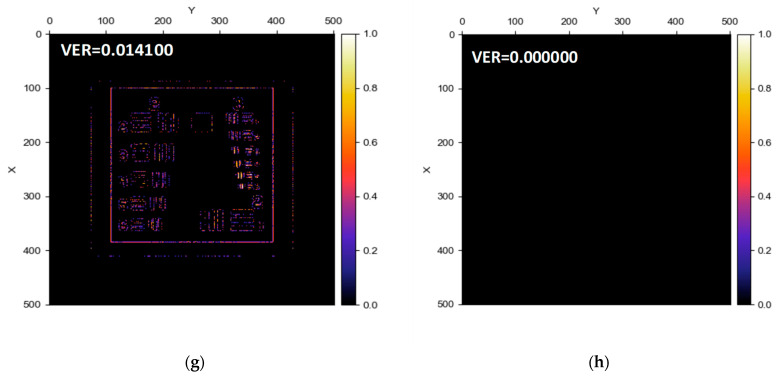
OSMO and EE OSMO performance comparison on ‘Reschart’: (**a**) VER; (**b**) PW; (**c**) IPDR; (**d**) EdgeE; (**e**) final dose histogram of OSMO; (**f**) final dose histogram of EE OSMO; (**g**) difference between target geometry and binary image of dose distribution using OSMO; and (**h**) difference between target geometry and binary image of dose distribution using EE OSMO.

Figure 11 shows the comparison curves of VER, PW, and IPDR of the ‘Reschart’ structure iterated by OSMO and EE OSMO, as well as the comparison images of the dose histogram. Table 1 shows the final iteration results of the four quality metrics. In the comparative experiment, the optimization parameters are $D_{h}\;$ = 0.9 and $D_{l}\;$ = 0.83, and the number of iterations is 30.

Figure 11a–d shows that EE OSMO has better convergence than OSMO. The quality metric values of 15 iterations of EE OSMO are comparable to those of 30 iterations of OSMO, while the former consumes only 2/3 of the time of the latter. After 30 iterations, the EE OSMO voxel error rate reaches 0 (VER = 0), there are no overlaps between the in-part histogram and out-of-part histogram (PW < 0), and the concentration of the dose distribution increased by 13%, and the edge’s error rate reaches 0 (EdgeE = 0) compared with the OSMO results, which are VER > 0, PW > 0, IPDR = 0.153547, and EdgeE = 0.00237, according to Figure 11e,f and Table 1.

Figure 11g is the difference image between the target geometry and binary image of the dose distribution, and the dose distribution is obtained with OSMO optimization. The binarization threshold of the dose distribution is the normalized minimum value of the dose distribution’s in-part voxels.

Figure 11h is the difference image between the target geometry and binary image of the dose distribution, and the dose distribution is obtained with EE OSMO optimization. The binarization threshold of the dose distribution is the normalized minimum value of the dose distribution’s in-part voxels.

From the comparison between Figure 11g,h, it can also be shown intuitively that the dose distribution simulation error of EE OSMO is close to 0, while the dose distribution simulation error with OSMO optimization is still significant.

**Figure 12:**
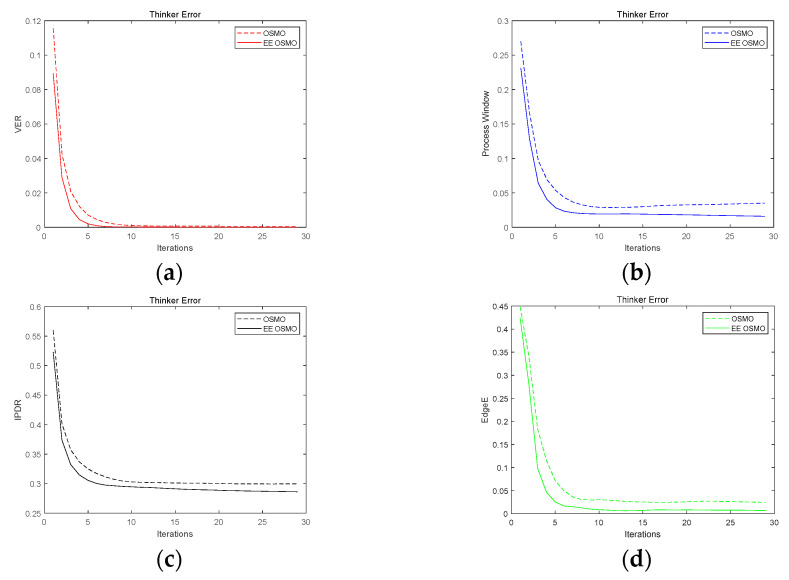
OSMO and EE OSMO performance comparison on ‘Thinker’: (**a**) VER; (**b**) PW; (**c**) IPDR; and (**d**) EdgeE.

The same conclusion is also verified by 3D image reconstruction. Figure 12 shows the comparison curves of VER, PW, and IPDR and EdgeE of the ‘Thinker’ structure iterated by OSMO and EE OSMO. Table 2 shows the final iteration results of the four quality metrics. In the comparative experiment, the optimization parameters are $D_{h}\;$ = 0.8 and $D_{l}\;$ = 0.6, and the number of iterations is 30.

The above two comparisons show that the results of EE OSMO are significantly improved in terms of convergence and optimization accuracy compared with OSMO.

**Figure 13:**
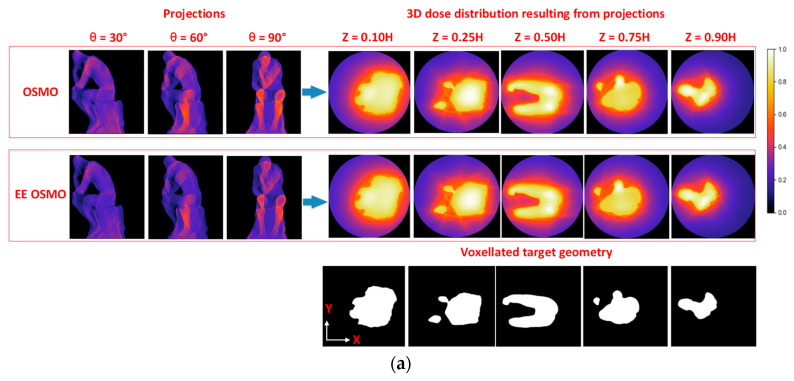

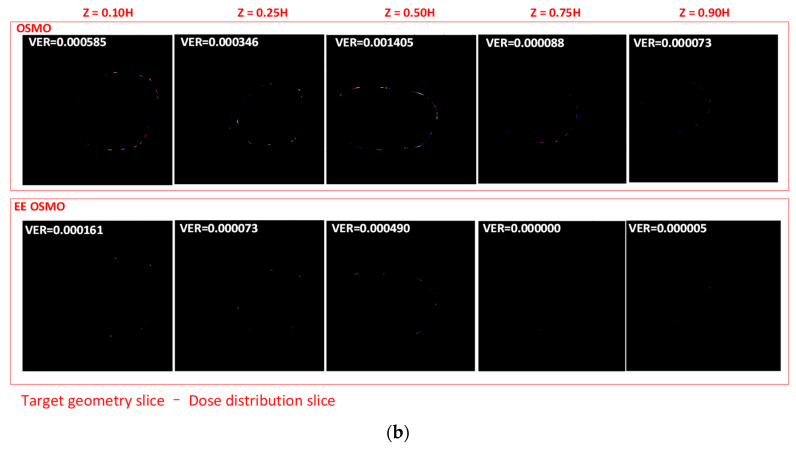
‘Thinker’ geometry reconstruction comparison between OSMO and EE OSMO: (**a**) computational projections and dose distribution slice comparison; (**b**) difference comparison between target geometry slices and dose distribution slices’ binary images.

Comparisons between OSMO and EE OSMO, including several projector intensity images, dose distribution slices for the ‘Thinker’ geometries, and difference images between target geometry slices and dose distribution slices’ binary images, are shown in Figure 13.

An the left of Figure 13a are three computational projections at 30°, 60° and 90°. An the top right of Figure 13a are five dose distribution slices with different heights. An the bottom right of Figure 13a are five target geometry slices with different heights, accordingly.

At the top of Figure 13b are the differences between target geometry slices and binary image of dose distribution slices, and the dose distribution slices are obtained with OSMO optimization. The binarization threshold of dose distribution is the normalized minimum value of the dose distribution’s in-part voxels.

A the bottom of Figure 13b are the differences between target geometry slices and binary image of dose distribution slices, and the dose distribution slices are obtained with EE OSMO optimization. The binarization threshold of dose distribution is the normalized minimum value of dose the distribution’s in-part voxels.

Figure 13a shows that the dose distributions obtained with OSMO and EE OSMO can both approximate the target geometry better, while the quantitative gap between them is difficult to distinguish by eye. In order to intuitively demonstrate the reconstruction quality gap between them further, the respective differences between target geometry slices and binary image of the dose distribution slices are calculated and shown in Figure 13b. From Figure 13b, it can be seen that there is a certain edge error in the OSMO’s dose distribution, while the edge error of EE OSMO’s is almost zero under the same parameters. After further analysis of Figure 13b, we found that the edge error of OSMO’s is nearly 3 voxels in certain locations. We assume to print a structure with a dimension of 1 cm^3^, and the spatial resolution of it is 512 × 512 × 512 voxels, that is, the size of one voxel is 19.5 × 19.5 × 19.5 μm. In the above case, the OSMO’s dose distribution error reaches nearly 60 μm in one dimension. That is to say, the larger the space occupied by a single voxel, the greater the error, and the quantitative gap between OSMO and EE OSMO is more significant.

### 5.2. The Influence of Frequency Filtering

To avoid the impossible negative light problem of frequency filtering during the iteration, the OSMO only performs frequency filtering in initialization to improve the rate of convergence of iterations, but it does not use it during the iteration. EE OSMO is the improved algorithm of OSMO, and its processing architecture is basically the same as OSMO. Therefore, in this section, only the error convergence performance difference between OSMO and EE OSMO is discussed, with and without frequency filtering and just in initialization.

The ’Reschart’ is selected as the geometry for performance comparison; the optimization parameters are $D_{h}\;$ = 0.9 and $D_{l\;}$ = 0.83, and the number of iterations is 30.

**Figure 14:**
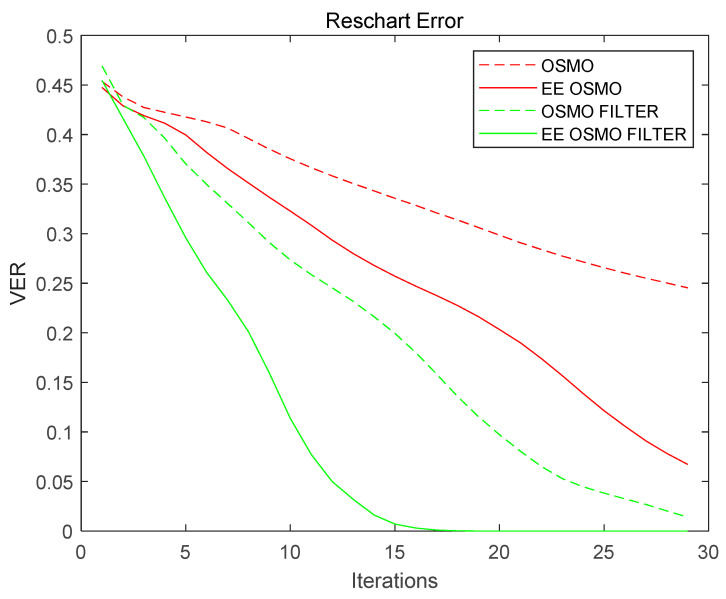
OSMO and EE OSMO performance comparison of VER on ‘Reschart’. The red curves represent VER iteration curves without frequency filtering initialization. The green curves represent VER iteration curves with frequency filtering initialization.

The two red curves in Figure 14 (dashed line for OSMO and solid line for EE OSMO) show the VER iteration curves of OSMO and EE OSMO with the same parameter but without frequency filtering initialization. For comparison, the two green curves in Figure 14 (dashed line for OSMO and solid line for EE OSMO) show the VER iteration curves of OSMO and EE OSMO with the same parameter and with frequency filtering initialization.

From the comparison of the red and green curves in Figure 14, it can be seen that under the same initialization conditions, EE OSMO has better convergence than OSMO. In addition, the target was frequency-filtered to generate an improved initial model. This reduces the number of algorithm iterations necessary to converge to an accurate solution compared with simply using the target as the initial model, and using this image as the initial iteration value has better convergence (as shown by the green curves).

## 6. Conclusions

We developed a new improved projection optimization algorithm in the image computation field of tomographic VAM, named ‘EE OSMO’. In this algorithm, some morphological processing was added to the optimization iteration of OSMO to improve the reconstruction accuracy of target boundaries (or edges). Accordingly, a new quality metric for volumetric printing, named ‘Edge Error’, is defined as the accuracy evaluation of target boundaries. The dose distribution was evaluated computationally. The EE OSMO approach was shown to perform better than OSMO in nearly all four computational metrics; thus, EE OSMO could achieve better convergence and higher accuracy.

## Figures and Tables

**Table 1:** Final iteration results on ‘Reschart’.

Algorithm	VER	PW	IPDR	EdgeE
OSMO	0.01410	0.03336	0.153547	0.00237
EE OSMO	0	−0.006759	0.12909	0

**Table 2:** Final iteration results on ‘Thinker’.

Algorithm	VER	PW	IPDR	EdgeE
OSMO	0.000578	0.035062	0.299614	0.024669
EE OSMO	0.000110	0.016018	0.286161	0.007178

## Data Availability

Not applicable.
